# Heat‐Treated Sucuk in Natural Casings: A Source of Higher Fermentative and Oxidative Volatile Compounds?

**DOI:** 10.1002/fsn3.70137

**Published:** 2025-04-18

**Authors:** Ahmet Dursun, Dilek Türkmen, Zehra Güler

**Affiliations:** ^1^ Faculty of Agriculture, Department of Food Engineering Hatay Mustafa Kemal University Antakya Turkey

**Keywords:** browning, casing, collagen, heat‐treatment

## Abstract

The effect of the type of casing on the quality parameters of Turkish‐style sausages (sucuk) is quite unexplored. So, this study aims to compare the effects of using natural or collagen casings in the production of naturally fermented (traditional) or heat‐treated sucuks on physicochemical properties (moisture, acidity, pH, and color values) and volatile compounds (VCs). The VCs, extracted by a solid‐phase‐micro‐extraction technique, were analyzed by gas chromatography‐mass spectrophotometry (SPME‐GC‐MS). Natural casing yielded a final product with the lowest pH, highest lightness, and redness in the traditional method, and the highest moisture and browning index in the heat‐treated method. Regardless of casing type, the traditional method produced sucuks with lower moisture, pH, a*, and browning index values. Cumin aldehyde, methyl eugenol, γ‐terpinene, pinene, and eugenol were the most abundant VCs identified in sucuk. The type of casing did not alter the volatile profile of naturally fermented sucuk. However, in heat‐treated sucuk, natural casing resulted in a higher accumulation of aldehydes, acids, and ketones in the final product compared to collagen casing. It is highlighted that natural casing may not be suitable for heat‐treated sucuk due to the abundance of lipid oxidation products. Principal component analysis showed that VCs were useful in differentiating between production stages and casing types, particularly for heat‐treated sucuk. This study demonstrates the impact of casing type on the quality of Turkish‐style sucuk and provides a theoretical basis for casing selection in future industrial production.

## Graphical abstract

The aim of this study was to determine the optimal casing material (natural or collagen) for use in the production of fermented or heat‐treated sucuk. Natural casing improved the products' redness but increased browning and oxidative‐derived volatile compounds in heat‐treated sucuk. In the heat‐treatment method, collagen casing caused greater moisture loss in sucuks compared to natural casing. Volatile compounds derived from the oxidation of amino acids (3‐methyl‐2‐butenal) or fatty acids (heptanal, trans‐2‐heptenal, nonanal, trans,trans‐2,4‐heptadienal, trans,trans‐2,4‐decadienal, trans,cis‐2,4‐decadienal) emerged in natural‐cased sucuks produced by heat treatment. Furthermore, ethanol, acetic acid, and acetoin derived mainly from carbohydrate and pyruvate metabolism were found in high abundance in natural‐cased products, particularly in heat‐treated sucuk. These findings offer valuable insights for the food industry, helping producers select the best casing material to optimize product quality. This research provides practical guidance for improving sucuk production processes and may interest food scientists and industry professionals, and consumers.

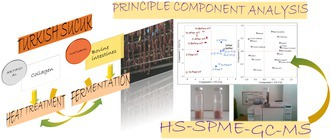

## 1 Introduction

Fermentation, drying, and salting have been the oldest methods of preserving food for centuries to ensure a long shelf life. These three methods are also used in the production of Turkish sausage (sucuk), known as a fermented meat product. In traditional sucuk production, the batter consisting mainly of beef meat, beef fat, salt, and various spices is stiffened in air‐dried bovine small intestines and is subjected to fermentation and drying (Kaban et al. 2022a). Fermentation is carried out by spontaneous microbiota in the traditional process and by commercial starter cultures in the industrial process (Kaban 2013). The product is sold in the Turkish market as “fermented sucuk.” However, recently, heat treatment has been included in the industrial‐scale production process of sucuk in order to reduce the production time. The product is presented to the market as “heat‐treated sucuk.” For heat‐treated sucuk, the fermentation process is typically very short, lasting only one night, followed by drying for about one night after heat treatment. After 3 to 5 days, the sucuk is ready to be sold for consumption. According to the Turkish Standard (TSI 2016), this product can contain high moisture compared to fermented sucuk (50% vs. 40% as maximum limit).

The choice of casing for sucuk production is a crucial step due to its direct and indirect role in volumetric, structural and chemical changes. The interactions between casing and filling during the production process determine the final product characteristics (Harper et al. 2012). In recent decades, the range of casing available to sausage (sucuk) producers has expanded considerably. In the past, “natural casings” from bovine small intestine were only used for sucuk production, whereas more recently “artificial casings” made from collagen and cellulose have been widely used for this purpose due to their low cost and also because they allow faster running speeds on manufacturing equipment (Feng et al. 2014). Collagen is the main component of bone, hide and connective tissue, which are by‐products of the meat industry. Therefore, collagen casings may contain fibrous and solubilized materials derived from these products (Ratanavaraporn et al. 2008). For this reason, they are comparable to natural casings.

In Turkey, both traditional and heat‐treated sucuks with natural or artificial casings are sold to meet consumer demand. However, traditionally produced sausage is generally preferred by consumers due to its sensory properties (Kaban and Kaya 2009). Both flavor, namely volatile and non‐volatile compounds, and appearance, namely texture and color, determine product acceptance. These quality attributes are mainly influenced by the raw material, production process, type of casing, etc. (Ensoy et al. 2010; Karslıoğlu et al. 2014; Montanari et al. 2016, 2022).

The volatile compounds are not only formed from the degradation of lipids, proteins, and carbohydrates during fermentation and ripening, but also directly from the spices used and the subsequent reactions between the formed volatile compounds and secondary biochemical events, such as the metabolism of fatty acids and amino acids (Ordonez et al. 1999; Kaban 2013; Tekin and Güler 2021). Volatile compounds such as hexanal and 2‐heptanal can be generated by the degradation of hydroperoxides derived from the oxidation of unsaturated fatty acids. They can give an idea of the enzymatic oxidation and auto‐oxidation of the lipids during the ripening process, such as the thiobarbituric acid reactive substrates (TBARS) value (Dursun and Güler 2023). Thus, volatile compounds are more informative about biochemical pathways during sucuk fermentation or ripening (Dursun and Güler 2019). The biochemical pathways and the course of chemical reactions are mainly dependent on technological processes, namely heat treatment, fermentation, and the type of casing, especially due to its permeability (Montanari et al. 2022).

In our previous study, there was a comparison of traditionally produced sucuks stuffed into poliamid‐polietilen plastic or natural casing in terms of volatile compounds (Dursun and Güler 2019). Yan et al. (2022) also reported that collagen casing improved the textural properties of fermented sausage, lowered pH and reduced biogenic amines, but had no significant effect on microbial growth and volatile compound formation. Montanari et al. (2022), comparing Italian fermented sausages stuffed into collagen and natural casing, reported that the use of collagen resulted in a greater reduction in pH and produced significant differences in flavor profile. Studies have mostly focused on the volatile compounds of sucuks sold on the market (Özkara et al. 2019; Kaban 2010) or sucuks using different starter cultures in production (Kaban and Kaya 2009; Öztürk et al. 2021; Kaban et al. 2022b), and also the microbial, physicochemical and proteolytic changes of traditionally produced and heat‐treated sucuks at different fermentation intervals (Dalmış and Soyer 2008; Ercoşkun et al. 2010; Bilenler et al. 2017). However, no data are available on the comparison of volatile compounds and color of “traditional” or “heat‐treated” sucuks stuffed in natural or artificial casings during the production process. Therefore, the aim of this study was to compare the physicochemical properties and volatile compounds of Turkish sucuks produced using two different production processes (traditional and heat treatment), and two types of sucuk casings (natural and collagen). Analyses were carried out at different stages of each production process in order to determine differences between the type of casings.

## 2 Material and Methods

### 2.1 Sucuk Production

The sucuk production was based on practices commonly used in a local butcher in Adana, Turkey. Sucuk formulation consisted of 80% beef meat, 20% sheep tail fat, 1.6% NaCl, 0.4% sucrose, 5% garlic, 1.6% red pepper, 1.6% hot red pepper, 1% black pepper, 1.2% cumin, 0.2% fenugreek, and 0.2% a spice mixture, including equally pimento, clove powder, cinnamon, ginger, and coriander seed powder.

After 24 h post‐slaughter, boneless rib meat was placed in a cutter (CTR100, Arı Makina, İstanbul, Turkey) the frozen beef fat was added; thereafter, the other ingredients were mixed. The prepared sucuk batter was conditioned at 4°C and about 68%–70% relative humidity (RH) for 24 h.

After sampling, the batter was separated into two equal parts. The first part and second part were stuffed into 38 mm artificial collagen casings and 40 mm natural casings from bovine intestines with a filling machine (UKMS‐32PT, Bosfor, İstanbul, Turkey), respectively. The sucuk coils weighed about 150 g and were re‐conditioned at 18°C–20°C for 6–8 h for moisture equilibration. Then each part was divided equally into two sub‐groups for traditional and heat treatment processes. Traditional groups were coded as NC‐T for natural casing and CC‐T for collagen casing. Similarly, heat treatment groups were coded as NC‐H for natural casing and CC‐H for collagen casing. In a climatic test cabinet (Qualitec, Biosan Food, Konya, Turkey), traditional sucuk groups were subjected to a gradual fermentation and drying process as follows: 1st process for 2 days at 24°C ± 1°C, 94% ± 4% RH under an air flow rate of 0.8–0.9 m/s, 2nd process for 2 days at 22°C ± 1°C, 84% ± 3% RH under an air flow rate of 0.6–0.7 m/s, and 3rd process for 2 days at 18°C ± 1°C, 77% ± 3% RH under an air flow rate of 0.4–0.5 m/s. After 3rd process, the sampling was done from sucuk as the final product.

In the heat‐treated sucuk production, after sampling from the sucuk coils kept at 4°C for 24 h, the sucuks were heated conventionally until the internal temperature of the sucuk coils reached 68°C (FPK 100, Arı Makina, Turkey) and cooled to 20°C–25°C with a cold water shower as stated by Armutcu et al. (2020). Following a two‐hour period of rest at 5°C, the sampling was conducted. Subsequently, the sucuks were hung‐dried for 12 h at ambient temperature (20°C–25°C), after which the sampling of the final product (after drying) was performed for analysis. The total processing time for the traditional and heat treatment sucuk was 7 and 4 days, respectively. During the production of both fermented and heat‐treated sucuk, the weight loss of samples was monitored to determine whether the moisture content fell below 40%.

### 2.2 Physicochemical Analysis

The moisture content of sucuk was determined gravimetrically by drying at 105°C (Method 950.46, AOAC 2000). Sucuk was homogenized with distilled water (1:10 *w*/*v*) and then measured using a pH meter (Orion, Thermo, Massachusetts, USA) calibrated with pH 4.0 and 7.0 buffers prior to measurement. The homogenate (10 mL) was titrated with 0.1 N NaOH in the presence of phenolphthalein indicator, and the titratable acidity was calculated as %lactic acid. Color analysis of sucuk samples was performed on the inner surface using a colorimeter (Hunter ColourFlex‐EZ, HunterLab, Virginia, USA) according to the CIE L* (lightness), a* (+/−: redness/greenness), b* (+/−: blueness/yellowness) system (Li et al. 2017). The browning index (BI) was calculated from the L*, a*, and b* values as described by Bozkurt and Bayram (2006) as follows:

$$\mathrm{BI}=\frac{\left[100\times \left(A-0.31\right)\right]}{0.17}$$

$$A=\frac{a^{*}+1.75\times L^{*}}{5.645\times L^{*}+a^{*}-3.012\times b^{*}}$$

### 2.3 Volatile Compounds Analysis

A solid phase microextraction fiber consisting of 50/30 μm divinylbenzene/carboxene/polydimethylsiloxane with a length of 1 cm (Supelco, Bellefonte, PA, USA) was used to adsorb VCs present in the headspace of the samples, as reported by Dursun and Güler (2019). The fiber, previously conditioned at 260°C for 30 min, was exposed to the headspace vials (20 mL) held in a water bath for 45 min at 60°C and held at the same temperature and time. The VCs were chromatographed on a HP‐Innowax capillary column (60 × 0.25 mm id × 0.25 μm film thickness) installed in a model 6890 GC coupled to a 5973 N MS (Agilent, Palo Alto, CA, USA). Helium was used as the carrier gas at a flow rate of 1 mL/min. GC‐MS conditions were established, and VCs were identified, as reported by Dursun and Güler (2019). The identification of VCs was performed by computer matching of their mass spectra against the Wiley7n.1 and NIST 02.L GC‐MS libraries. The results of the VC analysis were expressed as the percentage of each compound, calculated by dividing its peak area by the total integrated area of all identified peaks.

### 2.4 Data Analysis

Sucuk characteristics were subjected to a 2 × 2 factorial arrangement, carried out in a completely randomized design. The main factors were type of casing (natural and artificial) and type of production (fermented and heat‐treated). The model was:

$$Y_{\mathrm{ij}}=\mu +\alpha_{i}+\beta_{j}+{\alpha \beta }_{\mathrm{ij}}+\varepsilon_{\mathrm{ij}}$$

where, *Y*_*ij*_: parameter analyzed; *μ*: the mean value; *α*_*i*_: the effect of casing type; *β*_*j*_: the effect of production type; *αβ*_*ij*_ the interaction of casing and production type; *ε*_*ij*_: the residual error.

In addition, one‐way analysis of variance was used to compare the means of the production stages for each fermented and heat‐treated sucuk. The means of both sucuks (fermented vs. heat‐treated) and casing (natural vs. artificial) were analyzed by independent samples *t*‐test. Duncan's post hoc multiple comparison test was used to assess significantly different means (*p* < 0.05). The data obtained were analyzed using the SPSS statistical program (Version 22.0, IBM, USA). Cell‐plots and principal component analysis of VCs were performed using the JMP (version 13, SAS, USA).

## 3 Result and Discussion

### 3.1 Physicochemical Properties

Table 1 presents the moisture, titratable acidity, pH, and color values for the sucuk samples. The moisture content of all samples decreased significantly during the production process. The greatest moisture losses were observed after the 2nd fermentation/drying stage for traditional sucuks and after heat treatment for heat‐processed ones. Among the final products, the heat‐treated sucuk with natural casing had the highest moisture content at 38.34%. The traditional process yielded the final products with the lowest moisture content. This could be attributed to the significant decrease in pH at the advanced stages of production, as lower pH results in greater moisture loss (Ekici et al. 2015). It is well‐known that the water‐holding capacity of meat proteins diminishes as the pH approaches the isoelectric point. The type of casing did not affect moisture content in the traditional method, but it did in the heat‐treated method (Table 1). The heat‐treated final product with collagen casing had a lower moisture content than the one with natural casing. Nevertheless, the final moisture contents of all experimental sucuks were below the upper limits (< 40% for traditional fermented sucuk and < 50% for heat‐treated sucuk) specified in the Turkish Standard (TSI 2016). With regard to pH, the traditional production method reduced the initial pH from 5.41 to 5.03–5.12 within 7 days (Table 1). This finding was in accordance with the results of previous studies (Çiçek and Polat 2016; Bilenler et al. 2017). This is a crucial aspect to ensure product safety. In the heat treatment method, there was an increase in pH from the initial dough to before heat treatment, followed by a decrease thereafter. This may be due to proteolytic and/or incomplete fermentation activity of the indigenous flora, possibly resulting from storage at 4°C for 24 h. A similar finding was reported by Kaban et al. (2022b) for the control group without starter. It has also been reported that heat treatment can cause protein denaturation and the release of soluble components, leading to a decrease in the pH of sucuks (Kaban et al. 2022c). In addition, water evaporation during heat treatment may concentrate organic acids, further contributing to pH reduction, a finding supported by the increased titratable acidity observed after heat treatment in the present study (Table 1). The final products of the heat‐treated group exhibited a higher pH than those of the traditional sucuks (*p* < 0.001). The pH values obtained for all final products were lower than those (5.21 pH for fermented and 5.60 pH for heat‐treated) reported by Bilenler et al. (2017) and the maximum values (5.40 for fermented and 5.60 for heat‐treated) stipulated by the Turkish legal regulation (TFC 2019). However, Armutcu et al. (2020), who used a starter culture in the production of sucuk, reported a lower pH value (4.95) in the heat‐treated final product than that observed in our study. In this context, it should be emphasized that the use of the starter culture lowers the pH and increases the lactic acid content. The titration acidity also showed a significant increase in the production stages where the pH decreased (Table 1). It reached a maximum in the final products. Among them, traditional sucuk with natural casing had the highest acidity and the heat‐treated sucuk with collagen casing had the lowest (Table 1). This finding was in accordance with the pH (Table 1).

**TABLE 1:** The moisture, titratable acidity, pH, and color values of traditional and heat‐treated sucuks.

Processes	Casings	Process stage	Moisture (%)	TA (%)	pH	*L* *	*a* *	*b* *	BI
Traditional	Natural	Initial batter	47.81 ± 1.78^a^	0.76 ± 0.04^c^	5.41 ± 0.01^a^	51.2 ± 1.52^a^	9.75 ± 0.47^c^	20.6 ± 0.43	64.6 ± 1.21^c^
		After1st F/D	43.19 ± 1.34^b^	0.84 ± 0.04^c^	5.31 ± 0.01^b^	49.4 ± 0.57^a^	9.45 ± 0.37^c^	20.6 ± 0.83	67.0 ± 4.30^bc^
		After 2nd F/D	38.33 ± 1.01^c^	1.08 ± 0.09^b^	5.22 ± 0.01^c,y^	45.7 ± 1.24^b,x^	11.7 ± 0.52^b^	22.2 ± 1.41	83.8 ± 3.97^a^
		Final product	35.37 ± 0.56^d,Y^	1.34 ± 0.04^a,y^	5.03 ± 0.01^d,y,Y^	50.0 ± 0.04^a,x,X^	12.7 ± 0.50^a,x^	21.8 ± 1.54	74.5 ± 5.74^b^
	Collagen	Initial batter	47.81 ± 1.78^a^	0.76 ± 0.04^d^	5.41 ± 0.01^a^	51.2 ± 1.52^a^	9.75 ± 0.47^b^	20.6 ± 0.43^b^	64.6 ± 1.21^c^
		After1st F/D	41.10 ± 0.24^b^	0.85 ± 0.05^c^	5.30 ± 0.02^b^	49.7 ± 1.51^ab^	9.58 ± 0.38^b^	19.9 ± 0.23^b^	64.3 ± 1.31^c^
		After 2nd F/D	37.58 ± 0.26^c^	1.12 ± 0.05^b^	5.32 ± 0.01^b,x^	43.4 ± 0.52^c,y^	11.5 ± 0.37^a^	21.8 ± 0.75^a^	87.1 ± 3.98^a^
		Final product	35.69 ± 0.68^d^	1.43 ± 0.04^a,x,X^	5.12 ± 0.01^c,x,Y^	47.9 ± 0.12^b,y,X^	9.74 ± 0.24^b,y,Y^	20.3 ± 0.09^b^	69.1 ± 0.33^b,Y^
Heat treatment (HT)	Natural	Initial batter	47.81 ± 1.78^a^	0.76 ± 0.04^c^	5.41 ± 0.01^d^	51.2 ± 1.52^a^	9.75 ± 0.47^c^	20.6 ± 0.43	64.6 ± 1.21^b^
		Before HT	45.50 ± 0.06^b,x^	0.76 ± 0.05^c^	5.78 ± 0.01^c^	50.8 ± 0.29^ab^	11.2 ± 0.06^b^	20.4 ± 0.87^y^	66.8 ± 2.81^b,y^
		After HT	40.27 ± 0.20^c,x^	0.88 ± 0.00^b^	5.68 ± 0.01^b,y^	48.1 ± 0.14^c,x^	11.8 ± 0.49^b,y^	19.9 ± 0.79^y^	68.8 ± 3.60^b,y^
		Final product	38.34 ± 0.11^d,x,X^	1.30 ± 0.00^a,x^	5.55 ± 0.01^a,y,X^	49.0 ± 1.11^bc,x,X^	13.2 ± 0.21^a,x^	20.9 ± 0.22	76.0 ± 0.52^a,x^
	Collagen	Initial batter	47.81 ± 1.78^a^	0.76 ± 0.04^c^	5.41 ± 0.01^d^	51.2 ± 1.52^a^	9.75 ± 0.47^c^	20.6 ± 0.43^b^	64.6 ± 1.21^c^
		Before HT	41.79 ± 0.40^b,y^	0.76 ± 0.05^c^	5.76 ± 0.01^c^	50.5 ± 0.12^a^	10.9 ± 0.36^b^	23.1 ± 0.90^a,x^	75.6 ± 2.88^b,x^
		After HT	38.13 ± 1.49^c,y^	0.89 ± 0.08^b^	5.72 ± 0.01^b,x^	43.2 ± 1.33^c,y^	14.6 ± 0.17^a,x^	23.5 ± 0.51^a,x^	100 ± 5.96^a,x^
		Final product	36.24 ± 0.16^c,y^	1.21 ± 0.00^a,y,Y^	5.57 ± 0.01^a,x,X^	46.1 ± 0.11^b,y,Y^	10.6 ± 0.16^b,y,X^	20.5 ± 0.16^b^	74.5 ± 0.29^b,y,X^
Process (*A*)									
Traditional			40.86 ± 4.88	1.02 ± 0.26	5.27 ± 0.13	48.6 ± 2.78	10.5 ± 1.25	20.9 ± 1.08	71.9 ± 9.11
Heat treatment			41.99 ± 4.42	0.91 ± 0.21	5.61 ± 0.14	48.8 ± 2.86	11.5 ± 1.63	21.2 ± 1.34	73.9 ± 11.4
*p* value			ns	ns	***	ns	*	ns	*
Casing (*B*)									
Natural			42.08 ± 4.59	0.96 ± 0.24	5.42 ± 0.23	49.4 ± 3.20	11.2 ± 1.40	20.9 ± 1.05	70.8 ± 7.06
Collagen			40.77 ± 4.71	0.97 ± 0.24	5.45 ± 0.21	47.9 ± 3.30	10.8 ± 1.62	21.3 ± 1.36	75.0 ± 12.5
*p* value			ns	ns	ns	ns	ns	ns	*
*A × B*			ns	ns	ns	ns	ns	**	*

*Note:* The data were expressed as mean ± standard deviation. ^a–d^The mean values followed by different letters in the same row indicate significant differences (*p* < 0.05) between production stages. ^x,y^The significance (*p* < 0.05) between natural and collagen casing of each process at each production stage is shown. ^X,Y^The significance (*p* < 0.05) between final products of traditional and heat‐treated sucuks at each casing is shown.

Abbreviations: BI, browning index; F/D, fermentation/drying; ns, not significant (*p* > 0.05); TA, titratable acidity (g lactic acid/100 g).

* *p* < 0.05.

** *p* < 0.01.

*** *p* < 0.001.

Regarding the type of casing, the use of natural casing resulted in final products with a relatively low pH compared to collagen casing (Table 1). This finding was not consistent with the results reported by Yan et al. (2022) and Montanari et al. (2022), who observed a lower pH value in collagen casing sausage compared to natural casing. The discrepancy is likely attributed to differences in casing diameter, sausage formulation, or ripening conditions.

In all the experimental groups, the values of the color parameters L*, a*, and b* varied during production from 43.3 to 51.2, from 9.45 to 14.6, and from 19.9 to 23.5, respectively (Table 1). A significant decrease in L* was observed in the traditional group on day 5 (after 2nd fermentation/drying) and in the heat‐treated group on day 3 (after heat treatment). At these stages, the use of collagen casing resulted in a 16% reduction in L* compared to the natural casing. After the 5th and 3rd days, the L* value increased; however, the final products exhibited lower L* values than the initial batter. In considering the final products, it is evident that the type of casing used had a more pronounced impact on lightness than the sucuk production methods used. The collagen‐cased final products were observed to be darker in color than those with natural casings, which is consistent with the findings of Zajac et al. (2021). This may be related to the pore size of the casing, which may also be a factor in oxygen permeability. Reduced oxygen permeability has been shown to lead to increased metmyoglobin formation (Dursun and Güler 2023). When we also considered the production method, the traditional with natural casing and the heat‐treated with collagen casing final products had the highest and lowest L* values (50.0 and 46.1, respectively). The final product with the lowest L* also had the lowest redness.

**FIGURE 1:**
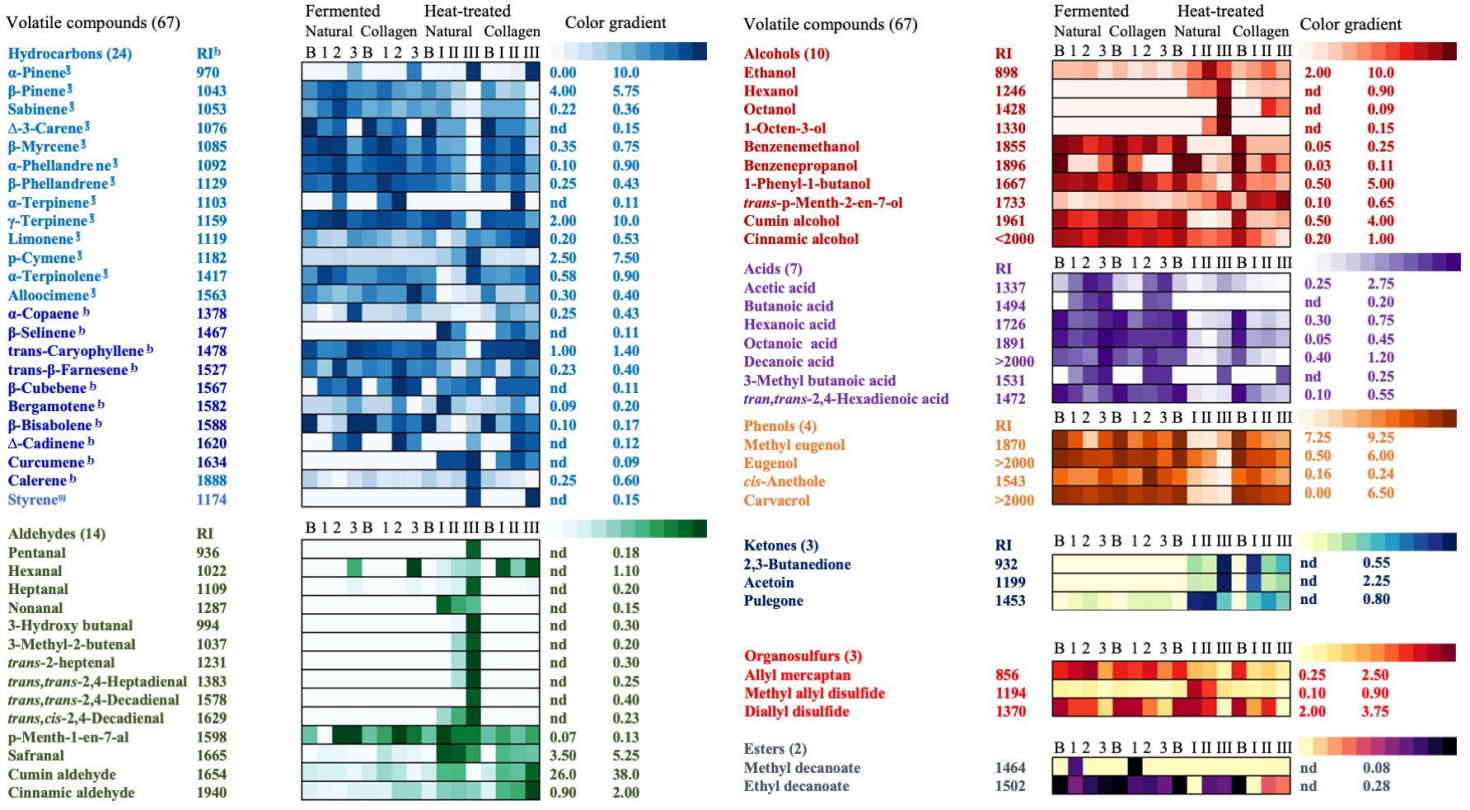
Cell‐plot for the volatile compounds identified in traditional and heat treated sucuks during the production stages (batter [B], 1st fermentation/drying [1], 2nd fermentation/drying [2], final product [3], and batter [B], before heat‐treatment [I], after heat treatment [II], final product [III], respectively). RI: Retention index based on identified compound retention times, calculated from the linear equation between each pair of straight alkane series (C8‐C20). nd, not detected.

The a* value indicates the intensity of the red color, and the mean value for heat‐treated sucuk was found to be high (Table 1). As reported by Liu et al. (2020), the growth of yeast in sausages can lead to an increase in redness due to its oxygen‐scavenging properties. This finding was supported by the presence of elevated levels of ethanol, the primary fermentation product produced by yeasts, in the heat‐treated sucuks (Figure 1). Furthermore, the elevated pH observed in the heat‐treated sucuks may indicate the presence of electron‐donating groups, such as amines, which could potentially lead to the reduction of metmyoglobin to deoxymyoglobin, thereby contributing to the observed increase in redness (Dursun and Güler 2023).

The highest level of redness was observed in collaged‐cased heat‐treated sucuk on day 3 (after heat treatment), followed by natural‐cased heat‐treated on day 4 (after drying), natural‐cased traditional on day 7 (after drying) and collagen‐cased traditional on day 5 (after 2nd fermentation/drying). As stated by Çiçek et al. (2015), the denaturation of myoglobin and oxymyoglobin due to high acidity and low pH results in a decrease in a* value, as observed in traditional sucuks.

Irrespective of the production methods used, the use of natural casing resulted in an increase in redness during the process, as evidenced by the high a* value observed in the final products (Table 1). The results demonstrate that the use of natural casing improves the red color stability. It is suggested that the processing methods, moisture loss, type of casing used, and pH are effective parameters influencing redness, possibly due to their effect on myoglobin forms and proportions in meat products. With regard to the final products, the type of casing had a more pronounced effect on redness.

A notable difference in yellowness (b*) was observed during processing when collagen casing was used, resulting in a relatively high b* value. Additionally, the interaction between casing type and processing method had a significant effect on yellowness (Table 1). However, neither the processing method nor the casing type affected the yellowness, irrespective of the sucuk production stages.

With regard to the browning index, the product with collagen casing had the highest browning index value after heat treatment, which was consistent with the low L* values observed. Bozkurt and Bayram (2006) have previously reported that a reduction in L* value is an indicator of dark coloring in sucuk, which is a consequence of the browning reaction. In terms of the final products, the highest browning index was observed in natural‐cased heat‐treated sucuk (Table 1).

Overall, the results of the present study showed lower L*, a*, and b* values than those reported by Ercoşkun et al. (2010) and Zajac et al. (2021), and lower a* and higher b* values than those reported by Armutcu et al. (2020). These differences may be related to the sucuk production protocol and casing type. The sucuk recipe did not include nitrite or nitrate, as consumer preferences have shifted towards additive‐free foods. Additionally, the starter culture was not used. The formation of nitrosomyoglobin due to the use of nitrate and/or nitrite and moisture loss is the main reason for the increase in a* values (Perez‐Alvarez et al. 1999). The low L* (dark color) and b* values are due to an increase in browning reactions. However, the L*, a*, b*, and BI values obtained in the present study were higher than those reported by Bozkurt and Bayram (2006), who observed an increase in a* value and a decrease in L* and b* in nitrate‐free sucuk during the first 5 days of ripening.

### 3.2 Volatile Compound (VC) Profile

A total of 67 volatile compounds (VCs) were identified in the headspaces of sucuk samples during production, including 24 hydrocarbons, 14 aldehydes, 10 alcohols, 7 acids, 4 phenols, 3 ketones, 3 organosulfurs, and 2 esters (Figure 1). The chemical groups found in sucuks were comparable to those reported by previous studies (Armutcu et al. 2020; Kaban and Kaya 2009; Kaban 2010; Sallan et al. 2022; Wang et al. 2022; Dursun and Güler 2019; Kaban et al. 2022b).

Production methodology, process stage, and casing type significantly influenced the volatile compound profile. The traditional group contained fewer VCs (50 out of 67 identified VCs) compared to the heat‐treated group. As can be seen in Figure 1, butanoic acid and methyl butanoate were identified only in the traditional group, whereas compounds such as hexanol, octanol, styrene, β‐selinene, curcumin, 1‐octen‐3‐ol, 2,3‐butanedione, acetoin, 3‐hydroxy butanal, 3‐methyl‐2‐butenal, trans‐2‐heptenal, nonanal, trans,trans‐2,4‐heptadienal, trans,trans‐2,4‐decadienal, trans,cis‐2,4‐decadienal were detected only in the heat‐treated group, indicating a notable impact of thermal processing on volatile composition. Among all samples, natural‐cased heat‐treated sucuk exhibited the highest number of volatile compounds (63 out of 67) (Figure 1). This is likely due to the higher permeability of natural casing to oxygen and moisture, which may have enhanced oxidative reaction products, resulting in a greater diversity of volatile compounds (Brody et al. 2001). It was consistent with the presence of higher levels of aldehydes and ketones formed by lipid oxidation in these samples (Figure 1).

In contrast with the findings of Yılmaz Oral and Kaban (2021), Kaban and Kaya (2009), who classified phenol‐containing compounds (eugenol, methyl eugenol) into the aromatic hydrocarbon (terpenes) category, as in the study reported by Corral et al. (2015), aldehydes and hydrocarbons were identified as the principal chemical groups in all the sucuks, based on both percentage composition and total number of VCs, respectively (Figure 1; Table 2). This distinction highlights possible methodological differences in compound classification and suggests that further investigations may be necessary to clarify the grouping of specific VCs in sucuk.

**TABLE 2:** Percentage composition of the groups of volatile compounds identified in traditional and heat‐treated sucuks.

Processes	Casings	Stages	Aldehydes	Hydrocarbons	Phenols	Alcohols	Organosulfurs	Acids	Ketones	Esters
Traditional	Natural	Initial batter	31.1 ± 0.49	24.4 ± 0.17^b^	21.4 ± 0.35^a^	13.4 ± 0.04^a^	5.69 ± 0.01^a^	3.68 ± 0.08^d^	nd	0.27 ± 0.00
		After1st F/D	32.2 ± 0.05^y^	25.2 ± 0.79^ab^	20.1 ± 0.35^ab^	12.3 ± 1.44^ab^	5.51 ± 0.18^a^	4.37 ± 0.10^c,x^	0.09 ± 0.02^b^	0.24 ± 0.05
		After 2nd F/D	31.8 ± 0.65^y^	26.3 ± 0.05^a,x^	18.1 ± 0.14^c,y^	12.5 ± 0.08^ab,x^	5.72 ± 0.13^a,x^	5.10 ± 0.24^b^	0.15 ± 0.01^a^	0.23 ± 0.02
		Final product	31.7 ± 0.37^y,Y^	28.1 ± 1.01^a^	19.5 ± 1.78^bc,X^	11.2 ± 0.11^b,x,X^	3.69 ± 0.66^b^	5.52 ± 0.32^a,X^	0.06 ± 0.00^c,y,Y^	0.26 ± 0.04
	Collagen	Initial batter	31.1 ± 0.49	24.4 ± 0.17	21.4 ± 0.35	13.4 ± 0.04	5.69 ± 0.01	3.68 ± 0.08	nd	0.27 ± 0.00
		After1st F/D	35.0 ± 0.41^x^	25.2 ± 0.19	19.4 ± 0.30	11.6 ± 0.26	5.52 ± 0.13	2.89 ± 0.07^y^	0.12 ± 0.02	0.29 ± 0.00
		After 2nd F/D	33.3 ± 0.17^x^	24.6 ± 0.03^y^	20.4 ± 0.55^x^	11.6 ± 0.02^y^	5.11 ± 0.16^y^	4.74 ± 0.21	0.13 ± 0.01	0.22 ± 0.01
		Final product	32.7 ± 0.23^x,Y^	29.5 ± 0.28	18.3 ± 0.49	10.1 ± 0.15^y,X^	3.92 ± 0.20^X^	5.20 ± 0.51^X^	0.13 ± 0.02^x,Y^	0.20 ± 0.01
Heat treatment (HT)	Natural	Initial batter	31.1 ± 0.49	24.4 ± 0.17	21.4 ± 0.35	13.4 ± 0.04	5.69 ± 0.01	3.68 ± 0.08	nd	0.27 ± 0.00
		Before HT	43.8 ± 0.16^x^	24.4 ± 0.06^x^	12.7 ± 0.25^y^	10.6 ± 0.11^x^	5.40 ± 0.14^x^	1.56 ± 0.21^y^	1.45 ± 0.10^y^	nd
		After HT	44.0 ± 1.85^x^	23.3 ± 1.56	11.3 ± 0.77^y^	13.3 ± 1.84^x^	4.82 ± 0.07^x^	1.87 ± 0.53	1.34 ± 0.22	0.20 ± 0.01^x^
		Final product	42.2 ± 0.85^x,X^	29.1 ± 0.93	9.20 ± 0.20^y,Y^	10.4 ± 0.44^x,Y^	2.76 ± 0.06	3.07 ± 0.25^x,Y^	3.10 ± 0.11^x,X^	0.19 ± 0.02^x^
	Collagen	Initial batter	31.1 ± 0.49	24.4 ± 0.17	21.4 ± 0.35	13.4 ± 0.04	5.69 ± 0.01	3.68 ± 0.08	nd	0.27 ± 0.00
		Before HT	39.4 ± 0.79^y^	23.3 ± 0.34^y^	19.2 ± 1.42^x^	9.90 ± 0.20^y^	3.62 ± 0.23^y^	2.05 ± 0.00^x^	2.56 ± 0.14^x^	nd
		After HT	38.4 ± 0.02^y^	24.8 ± 0.96	18.3 ± 1.49^x^	11.1 ± 0.39^y^	4.37 ± 0.12^y^	1.76 ± 0.21	1.19 ± 0.22	0.10 ± 0.01^y^
		Final product	39.4 ± 1.04^y,X^	29.5 ± 0.48	16.9 ± 1.12^x^	8.60 ± 0.48^y,Y^	2.65 ± 0.10^Y^	1.64 ± 0.00^y,Y^	1.27 ± 0.00^y,X^	0.08 ± 0.01^y^
Process (*A*)										
Traditional			32.4 ± 1.27	26.0 ± 1.84	19.8 ± 1.33	12.0 ± 1.19	5.10 ± 0.82	4.40 ± 0.90	0.09 ± 0.06	0.25 ± 0.04
Heat treatment			38.7 ± 4.93	25.4 ± 2.45	16.3 ± 4.53	11.3 ± 1.85	4.37 ± 1.19	2.41 ± 0.89	1.36 ± 1.04	0.14 ± 0.11
*p* value			***	ns	***	ns	*	***	***	***
Casing (*B*)										
Natural			36.0 ± 5.87	25.7 ± 2.05	16.7 ± 4.71	12.1 ± 1.39	4.91 ± 1.08	3.60 ± 1.37	0.77 ± 1.07	0.21 ± 0.09
Collagen			35.0 ± 3.44	25.7 ± 2.31	19.4 ± 1.69	11.2 ± 1.64	4.57 ± 1.08	3.21 ± 1.30	0.68 ± 0.89	0.18 ± 0.10
*p* value			ns	ns	**	*	ns	ns	ns	ns
*A × B*			*	ns	**	ns	ns	ns	ns	ns

*Note:* The data were expressed as mean ± standard deviation. ^a–d^The mean values followed by different letters in the same row indicate significant differences (*p* < 0.05) between production stages. ^x,y^The significance (*p* < 0.05) between natural and collagen casing of each process at each production stage was shown. ^X,Y^The significance (*p* < 0.05) between traditional and heat‐treated sucuks at the mean values of each casing was shown.

Abbreviations: F/D, fermentation/drying; nd, not detected; ns, not significant (*p* > 0.05).

* *p* < 0.05.

** *p* < 0.01.

*** *p* < 0.001.

The abundance of volatile compound groups, with the exception of hydrocarbons, was significantly influenced by the production method used. Heat treatment yielded a high percentage of aldehydes and ketones, whereas the traditional process resulted in the production of esters and alcohols. The type of casing also had a significant effect on the levels of phenols and alcohols, with higher levels observed in collagen‐ and natural‐cased sucuks, respectively (Table 2).

Only 17 of the 67 VCs were identified at levels above 1% at any stage of sucuk production, representing approximately 88% of the total VCs identified in terms of percentage composition (Table 3). Thus, a total of 17 VCs were considered to reveal the effects of the production method and type of casing on volatile compounds.

**TABLE 3:** The mean relative percentage composition of the major volatile compounds in traditional and heat‐treated sucuks.^e^

Processes	Casings	Stages	Cumin aldehyde	Safranal	Cinnamic aldehyde	γ‐Terpinene	β‐pinene	α‐Pinene	p‐Cymene	Methyl eugenol	Eugenol
Traditional	Natural	Initial batter	26.0 ± 0.48^c^	3.92 ± 0.09	1.09 ± 0.07^a^	9.00 ± 0.04^c^	5.10 ± 0.04^b^	0.25 ± 0.09^b^	3.63 ± 0.04b	9.19 ± 0.09^a^	5.87 ± 0.20^a^
		After1^st^ F/D	27.3 ± 0.19^a,y^	3.80 ± 0.02	1.01 ± 0.07^ab^	9.47 ± 0.41^b^	5.43 ± 0.05^a^	0.35 ± 0.00^b^	3.64 ± 0.11b	8.62 ± 0.15^a^	5.49 ± 0.07^ab^
		After 2nd F/D	26.9 ± 0.51^ab,y^	3.82 ± 0.17	0.95 ± 0.05^b,y^	9.92 ± 0.03^a,x^	5.53 ± 0.02^a,x^	0.40 ± 0.03^b^	3.83 ± 0.01^a,x^	7.67 ± 0.08^b,y^	4.93 ± 0.01^b,y^
		Final product	26.5 ± 0.34^bc,y,Y^	3.68 ± 0.05	1.10 ± 0.02^a,y,Y^	8.35 ± 0.09^d,X^	5.13 ± 0.16^b,X^	4.56 ± 0.78^a,Y^	3.62 ± 0.08^b,Y^	8.70 ± 0.91^a^	4.80 ± 0.35^b,X^
	Collagen	Initial batter	26.0 ± 0.48^c^	3.92 ± 0.09^c^	1.09 ± 0.07^b^	9.00 ± 0.04^a^	5.10 ± 0.04	0.25 ± 0.09^b^	3.63 ± 0.04	9.19 ± 0.09^a^	5.87 ± 0.20^a^
		After1st F/D	29.6 ± 0.47^a,x^	4.17 ± 0.03^a^	1.08 ± 0.03^b^	9.32 ± 0.10^a^	5.35 ± 0.02	0.31 ± 0.02^b^	3.66 ± 0.03	8.33 ± 0.19^c^	5.32 ± 0.01^b^
		After 2nd F/D	27.8 ± 0.20^b,x^	4.03 ± 0.02^b^	1.31 ± 0.06^a,x^	8.99 ± 0.03^a,y^	5.00 ± 0.05^y^	0.36 ± 0.06^b^	3.47 ± 0.04^y^	8.79 ± 0.28^b,x^	5.47 ± 0.06^b,x^
		Final product	27.3 ± 0.12^b,x,Y^	3.63 ± 0.04^d^	1.30 ± 0.02^a,x,Y^	7.90 ± 0.65^b,X^	4.87 ± 0.36^X^	7.05 ± 2.13^a,Y^	3.56 ± 0.18	8.46 ± 0.05^bc^	4.09 ± 0.23^c,X^
Heat treatment (HT)	Natural	Initial batter	26.0 ± 0.48^c^	3.92 ± 0.09^b^	1.09 ± 0.07^b^	9.00 ± 0.04^b^	5.10 ± 0.04^a^	0.25 ± 0.09^b^	3.63 ± 0.04^c^	9.19 ± 0.09^a^	5.87 ± 0.20^a^
		Before HT	37.4 ± 0.26^a,x^	4.51 ± 0.03^a^	1.37 ± 0.07^b,y^	9.33 ± 0.08^a^	4.85 ± 0.06^ab,x^	0.32 ± 0.01^b^	4.15 ± 0.03^c,x^	7.48 ± 0.20^bc^	3.84 ± 0.06^b,y^
		After HT	37.1 ± 1.70^a,x^	4.53 ± 0.18^a^	1.40 ± 0.10^a,y^	7.48 ± 0.11^c,y^	4.57 ± 0.29^b^	0.53 ± 0.13^b^	5.18 ± 0.77^b,x^	7.41 ± 0.21^c^	2.85 ± 0.43^c,y^
		Final product	34.3 ± 0.28^b,x,X^	3.50 ± 0.39^b^	1.35 ± 0.06^a,y,X^	2.91 ± 0.25^d,y,Y^	4.02 ± 0.15^c,Y^	9.75 ± 0.75^a,X^	7.27 ± 0.15^a,x,X^	7.86 ± 0.31^b^	0.54 ± 0.05^d,y,Y^
	Collagen	Initial batter	26.0 ± 0.48^c^	3.92 ± 0.09	1.09 ± 0.07^c^	9.00 ± 0.04^a^	5.10 ± 0.04^a^	0.25 ± 0.09^c^	3.63 ± 0.04^a^	9.19 ± 0.09^a^	5.87 ± 0.20^a^
		Before HT	33.0 ± 0.68^a,y^	4.49 ± 0.16	1.54 ± 0.04^b,x^	8.67 ± 0.24^a^	4.55 ± 0.05^b,y^	0.32 ± 0.00^c^	3.32 ± 0.07^b,y^	8.47 ± 0.65^ab^	4.77 ± 0.35^b,x^
		After HT	32.2 ± 0.10^ab,y^	4.39 ± 0.09	1.60 ± 0.01^b,x^	8.71 ± 0.25^a,x^	4.69 ± 0.17^b^	0.85 ± 0.34^b^	3.55 ± 0.22^a,y^	8.08 ± 0.63^b^	4.62 ± 0.39^b,x^
		Final product	31.9 ± 0.40^b,y,X^	5.07 ± 0.86	1.99 ± 0.17^a,x,X^	6.33 ± 0.11^b,x,Y^	4.09 ± 0.03^c,Y^	9.90 ± 0.11^a,X^	2.99 ± 0.02^c,y^	7.95 ± 0.60^b^	3.52 ± 0.24^c,x,Y^
Process (*A*)											
Traditional			27.2 ± 1.16	3.87 ± 0.18	1.12 ± 0.13	8.99 ± 0.65	5.19 ± 0.25	1.69 ± 2.60	3.63 ± 0.12	8.62 ± 0.55	5.23 ± 0.59
Heat treatment			32.2 ± 4.20	4.29 ± 0.55	1.43 ± 0.29	7.68 ± 2.07	4.62 ± 0.41	2.77 ± 4.17	4.22 ± 1.36	8.20 ± 0.75	3.98 ± 1.69
*p* value			***	***	***	**	***	ns	*	*	***
Casing (*B*)											
Natural			30.2 ± 4.93	3.96 ± 0.38	1.17 ± 0.18	8.18 ± 2.16	4.97 ± 0.48	2.05 ± 3.31	4.37 ± 1.25	8.26 ± 0.78	4.27 ± 1.76
Collagen			29.2 ± 2.74	4.20 ± 0.50	1.37 ± 0.31	8.49 ± 0.95	4.85 ± 0.40	2.41 ± 3.71	3.48 ± 0.23	8.56 ± 0.56	4.94 ± 0.83
*p* value			ns	*	***	ns	ns	ns	***	ns	ns
*A × B*			*	ns	ns	ns	ns	ns	***	ns	*

Processes	Casings	Stages	Carvacrol	Ethanol	1‐Phenyl‐1‐butanol	Cumin alcohol	Diallyl disulfide disulphide	Allyl mercaptan	Acetic acid	Acetoin
Traditional	Natural	Initial batter	6.12 ± 0.03^a^	3.89 ± 0.15	4.48 ± 0.06^a^	3.61 ± 0.04^a^	3.59 ± 0.11^a^	1.89 ± 0.10^b^	1.01 ± 0.26^c^	nd
		After1^st^ F/D	5.81 ± 0.14^ab^	3.88 ± 1.17	4.27 ± 0.42^a^	2.83 ± 0.02^c^	3.17 ± 0.39^a^	2.15 ± 0.20^ab^	1.78 ± 0.02^b,x^	nd
		After 2nd F/D	5.33 ± 0.04^b,y^	4.13 ± 0.07^x^	4.55 ± 0.20^a^	2.71 ± 0.07^c^	3.17 ± 0.04^a,x^	2.34 ± 0.07^a,x^	2.54 ± 0.09^a,x^	nd
		Final product	5.80 ± 0.53^ab,X^	2.85 ± 0.46^Y^	3.52 ± 0.14^b,x,X^	3.40 ± 0.19^b,X^	2.38 ± 0.34^b^	1.19 ± 0.30^c,X^	2.32 ± 0.52^ab,X^	nd
	Collagen	Initial batter	6.12 ± 0.03^a^	3.89 ± 0.15^a^	4.48 ± 0.06^b^	3.61 ± 0.04^a^	3.59 ± 0.11^a^	1.89 ± 0.10^ab^	1.01 ± 0.26^b^	nd
		After1^st^ F/D	5.53 ± 0.10^b^	2.73 ± 0.07^c^	4.93 ± 0.09^a^	2.74 ± 0.05^c^	3.57 ± 0.02^a^	1.78 ± 0.15^b^	0.62 ± 0.03^b,y^	nd
		After 2nd F/D	5.88 ± 0.20^a,x^	3.44 ± 0.05^b,y^	4.22 ± 0.13^c^	2.65 ± 0.11^c^	2.95 ± 0.11^b,y^	2.00 ± 0.03^a,y^	1.82 ± 0.04^a,y^	nd
		Final product	5.51 ± 0.21^b^	2.59 ± 0.06^c,Y^	3.10 ± 0.06^d,y,X^	3.23 ± 0.10^b,X^	2.38 ± 0.24^c^	1.41 ± 0.05^c,X^	2.35 ± 0.61^a,X^	nd
Heat treatment (HT)	Natural	Initial batter	6.12 ± 0.03^a^	3.89 ± 0.15^c^	4.48 ± 0.06^a^	3.61 ± 0.04^a^	3.59 ± 0.11^a^	1.89 ± 0.10^a^	1.01 ± 0.26^ab^	nd
		Before HT	1.23 ± 0.01^b,y^	5.70 ± 0.11^b,x^	2.75 ± 0.11^b^	0.75 ± 0.03^c,y^	3.56 ± 0.01^a,x^	1.03 ± 0.11^b,x^	0.39 ± 0.01^c^	0.54 ± 0.14^b,y^
		After HT	0.87 ± 0.12^c,y^	9.24 ± 1.68^a,x^	1.97 ± 0.09^c,y^	0.55 ± 0.01^d,y^	3.29 ± 0.13^b^	0.90 ± 0.19^b^	0.81 ± 0.48^bc^	0.45 ± 0.22^b^
		Final product	0.48 ± 0.09^d,y,Y^	6.47 ± 0.45^b,x,X^	0.76 ± 0.05^d,y,Y^	0.86 ± 0.01^b,y,Y^	2.04 ± 0.03^c^	0.46 ± 0.04^c,Y^	1.36 ± 0.06^a,x,Y^	2.21 ± 0.06^a,x^
	Collagen	Initial batter	6.12 ± 0.03^a^	3.89 ± 0.15^c^	4.48 ± 0.06^a^	3.61 ± 0.04^a^	3.59 ± 0.11^a^	1.89 ± 0.10^a^	1.01 ± 0.26^a^	nd
		Before HT	5.77 ± 0.44^ab,x^	4.80 ± 0.12^b,y^	2.28 ± 0.04^b^	1.29 ± 0.06^c,x^	2.74 ± 0.10^c,y^	0.71 ± 0.12^c,y^	0.46 ± 0.08^b^	1.70 ± 0.07^a,x^
		After HT	5.36 ± 0.48^b,x^	6.06 ± 0.51^a,y^	2.40 ± 0.12^b,x^	1.24 ± 0.03^c,x^	3.26 ± 0.11^b^	0.89 ± 0.00^b^	0.32 ± 0.03^b^	0.53 ± 0.15^b^
		Final product	5.10 ± 0.30^b,x^	4.14 ± 0.31^c,y,X^	1.64 ± 0.07^c,x,Y^	1.52 ± 0.11^b,x,Y^	2.02 ± 0.09^d^	0.52 ± 0.00^d,Y^	0.27 ± 0.01^b,y,Y^	0.69 ± 0.09^b,y^
Process (*A*)										
Traditional			5.76 ± 0.33	3.43 ± 0.70	4.19 ± 0.59	3.10 ± 0.40	3.10 ± 0.51	1.83 ± 0.38	1.68 ± 0.74	nd
Heat treatment			3.88 ± 2.43	5.52 ± 1.81	2.59 ± 1.25	1.68 ± 1.18	3.01 ± 0.64	1.04 ± 0.54	0.70 ± 0.42	0.77 ± 0.76
*p* value			***	***	***	***	ns	***	***	***
Casing (*B*)										
Natural			3.97 ± 2.48	5.01 ± 2.07	3.35 ± 1.35	2.29 ± 1.28	3.10 ± 0.58	1.48 ± 0.66	1.40 ± 0.76	0.40 ± 0.73
Collagen			5.67 ± 0.41	3.94 ± 1.09	3.44 ± 1.19	2.48 ± 0.96	3.01 ± 0.58	1.39 ± 0.57	0.98 ± 0.75	0.37 ± 0.58
*p* value			***	**	ns	ns	ns	ns	*	ns
*A × B*			***	ns	ns	ns	ns	ns	ns	ns

*Note:* The data were expressed as mean ± standard deviation. ^a–d^The mean values followed by different letters in the same row indicate significant differences (*p* < 0.05) between production stages. ^x,y^The significance (*p* < 0.05) between natural and collagen casing of each process at each production stage is shown. ^X,Y^The significance (*p* < 0.05) between final traditional and heat‐treated sucuks of each casing is shown.

Abbreviations: F/D, fermentation/drying; nd, not detected; ns, not significant (*p* > 0.05).

^e^ The volatile compounds that had a relative percent value higher than 1% were considered major volatile compounds.

* *p* < 0.05.

** *p* < 0.01.

*** *p* < 0.001.

Cumin aldehyde was the major aldehyde and also VC identified in all the samples during production, followed by safranal and cinnamic aldehyde. It is derived from cumin, which is used in the production of sucuk (Ağaoğlu et al. 2007). In contrast to cinnamic aldehyde, which is the main compound in cinnamon used in current sucuks, cumin aldehyde belongs to the benzaldehyde class and is a common compound identified in Turkish sucuks (Kaban and Kaya 2009; Kaban 2010; Öztürk et al. 2021). With decreasing moisture content, cumin aldehyde increased significantly after the 1st fermentation/drying and before heat treatment compared to the initial batter. However, the traditional process gave a low mean compared to the heat treatment regardless of the process steps (Table 3). This can be explained by the long‐term fermentation which allows the reduction of aldehydes to alcohols in the traditional method. Regardless of the production method, the type of casing had no effect on cumin aldehyde. However, the interaction between the type of casing and the production process did have an impact. Additionally, the levels of safranal and cinnamic aldehydes were found to be significantly influenced by the type of casing and the production method. These compounds were the most abundant in the collagen‐cased heat‐treated final product (Table 3). Kaban and Kaya (2009) reported 2‐methyl‐3‐phenyl‐propanal, a member of cinnamic aldehydes, as an important aldehyde for sucuk.

With regard to hydrocarbons, our findings indicate that terpenes, particularly monoterpenes, are the second most abundant chemical group. This outcome differs from those reported by Kaban and Kaya (2009) and Kaban (2010), who identified terpenes and acids in sucuks of various brands, and terpenes in sucuks without starter and fermented with *Lactobacillus plantarum* and *Staphylococcus xylosus* as the primary volatile compounds. The main source of terpenes in sucuk production is spices such as cumin, black pepper, pimento, and paprika (Kaban et al. 2022b). The total terpenes level increased significantly throughout the production process, reaching a maximum in the final product. This is likely due to the increased dry matter content. Misharina et al. (2001) reported that terpenes increased with ripening time. The total terpene level in the final products was not influenced by the production process or the casings used (Table 2). However, collagen casing increased the percentage of sesquiterpenes and decreased the percentage of monoterpenes. This may be related to the fact that microbial enzymes play an important role in sesquiterpene synthesis depending on the sucuk medium (Degenhardt et al. 2009). Among the present terpenes, γ‐terpinene, α‐pinene, β‐pinene, and p‐cymene were the four most abundant monoterpenes contributing to the flavor of sucuk. Among them, γ‐terpinene was the second most abundant terpene in all groups during the production process, except for heat‐treated final products, where α‐pinene exhibited considerably higher levels (Table 3). Notably, γ‐terpinene showed the greatest decrease in the heat‐treated natural‐cased final product compared to the other final products, while cymene reached a maximum value. This may be attributed to degradation of γ‐terpinene to p‐cymene, and/or the leaching out of γ‐terpinene together with fat during heat treatment, as terpenes are oil‐soluble compounds. Additionally, α‐pinene exhibited a notable increase exclusively during sucuk production, reaching a maximum level in the final products of all groups. A greater increase in α‐pinene was observed in heat‐treated sucuks (Table 3). This may be attributed to microbial synthesis of α‐pinene in relation to the pH of the medium, as some bacteria, such as *Corynebacterium glutamicum* , are known to produce α‐pinene (Niu et al. 2018).

*p*‐Cymene and γ‐terpinene have previously been identified in traditional sucuk samples (Kaban 2010; Öztürk et al. 2021) and sucuks produced with *L. plantarum* and *S. xylosus* or with lactic acid bacteria (Kaban and Kaya 2009), and also *p*‐cymene only in traditional Spanish dry fermented sausage as the dominant terpene (Corral et al. 2015).

The current study demonstrates that the abundance of individual terpenes is closely related to the sucuk production process and the type of casing used. Notable shifts in terpenes were observed in the heat‐treated final products, with γ‐terpinene becoming the dominant compound instead of α‐pinene. Among the principal terpenes, β‐pinene was significantly higher in traditional sucuks, particularly in natural casing, compared to the heat‐treated sample (Table 3). γ‐Pinene (27.4%) and p‐cymene (20.49%) are important constituents of cumin essential oil (Viuda‐Martos et al. 2007).

Phenolic compounds were the third most prevalent chemical group identified in sucuk samples. The total phenol percentage showed a tendency to decrease throughout the sucuk production process, as shown in Table 2. A more pronounced decrease was observed in the heat‐treated process compared to the traditional one. In particular, the total phenol percentage of heat‐treated sucuk in natural casing decreased from 21% in the initial batter to 9% in the final product after drying. The type of casing did not affect the total phenol proportion in the traditional process; however, it did in the heat‐treated process. All the identified phenols, with the exception of cis‐anethole, were the principal compounds detected above 1% (Table 3). Methyl eugenol was the most abundant phenol, with a slight decrease observed during sucuk production, which was not influenced by the type of casing. However, eugenol and carvacrol demonstrated a notable decrease throughout the heat treatment, particularly in the natural‐cased sucuk. This may be attributed to the formation of eugenol derivatives, which are dependent on temperature and the availability of oxygen due to the pore size of the casing. Both eugenol and its methyl ether (methyl eugenol) are mainly derived from cloves and also from pimento (Padmakumari et al. 2011). In contrast to methyl eugenol, eugenol was predominantly identified in fermented sausages (Kaban and Kaya 2009; Kaban 2010; Öztürk et al. 2021; Yan et al. 2022). A previous study (Corral et al. 2015) revealed that methyl eugenol was present at higher levels than eugenol in Spanish traditional dry‐fermented sausages, as in the present study. The elevated levels of methyl eugenol compared to eugenol in the sausages may be related to the source of the clove or pimento used, as pimento from China and Mexico has been shown to contain high levels of methyl eugenol (Avila et al. 2022). It has been documented that eugenol and methyl eugenol are the main contributors to the “tallowy,” “spicy,” and “clover” flavors observed in sausages (Corral et al. 2015). To the best of our knowledge, carvacrol has only been identified in one study (Öztürk et al. 2021). Like cuminaldehyde and safranal, carvacrol can be derived from cumin, which is used in the production of sucuk (Ağaoğlu et al. 2007; Li and Jiang 2004).

Among the alcohol compounds, ethanol was significantly (*p* < 0.001) higher in heat‐treated sucuk than in traditional sucuk, regardless of the types of casing used (Table 3). However, 1‐phenyl‐1‐butanol and cumin alcohol were found at high levels in traditional sucuk. Regardless of the production method, the ethanol percentage increased significantly in natural casing. However, the type of casing had no effect on 1‐phenyl‐1‐butanol and cumin alcohol. The highest ethanol was observed in the natural casing product after heat treatment. It is possible that the combination of relatively high pH and temperature during the heat treatment process accelerates alcohol fermentation. Conversely, high levels of eugenol in traditional sucuks may inhibit dehydrogenase activity, which is essential for ethanol production (Nejad et al. 2017). Kaban et al. (2022b) reported that the biodiversity of the spontaneous microflora in starter‐free sucuks increased in ethanol during production. In contrast to ethanol, acetic acid, the most prevalent acid, demonstrated a consistent increase throughout the traditional sucuk production process. However, the application of heat treatment resulted in a decrease in acetic acid, as reported by Çakır et al. (2013). It seems that acetic acid fermentation does not occur in the heat‐treated groups during production. Regardless of the production method, natural casing significantly (*p* < 0.05) enhanced acetic acid production compared to collagen casing. Acetic acid has been identified by Kaban and Kaya (2009) and Kaban (2010) in dry fermented sausages and has also been reported as the primary acid in Italian fermented sausage (Montanari et al. 2022). This compound is mainly produced by lactic acid bacteria and staphylococci during carbohydrate metabolism. It is also formed by lipid oxidation and amino acid catabolism (Montel et al. 1998; Montanari et al. 2022).

Allyl mercaptan and diallyl disulfide are organosulfur compounds derived from garlic used for sucuk production. The latter compound was not affected by the production method and type of casing. However, while allyl mercaptan decreased during the heat treatment process, it increased during the traditional process and reached a maximum on 2nd fermentation/drying (Table 3). It has been reported that lactic acid bacteria, such as *L. plantarum* , produce allyl mercaptan from diallyl disulfide in garlic‐ or onion‐enriched foods during fermentation (Jo et al. 2020). The notable decrease in allyl mercaptan in heat‐treated final sucuks compared to the initial batter can be attributed to both non‐production and evaporation during the process.

During the production of sucuk, esters and ketone chemical groups were detected at low levels. Furthermore, the ester compounds were not identified in the heat‐treated sucuks before heat treatment, and the ketones were not present in the sucuk batter or in the traditional sucuks. There was a notable increase in esters with the traditional process, while the heat treatment process resulted in a significant increase in ketones. However, the increase in ketones in heat‐treated sucuk did not follow a consistent pattern during production. The type of casing had no impact on the total ketone and ester levels. Of the ketones, acetoin was the most abundant ketone, reaching a maximum in heat‐treated sucuk with natural casing at the final stage, but before heat treatment in sucuk with collagen casing. This ketone, which is responsible for the buttery flavor, was previously identified in heat‐treated sucuk (Sallan et al. 2022). As noted by Dursun and Güler (2019), acetoin production may be closely related to the availability of Enterococcus and the internal pH of sucuk.

### 3.3 Principle Component Analysis

**FIGURE 2:**
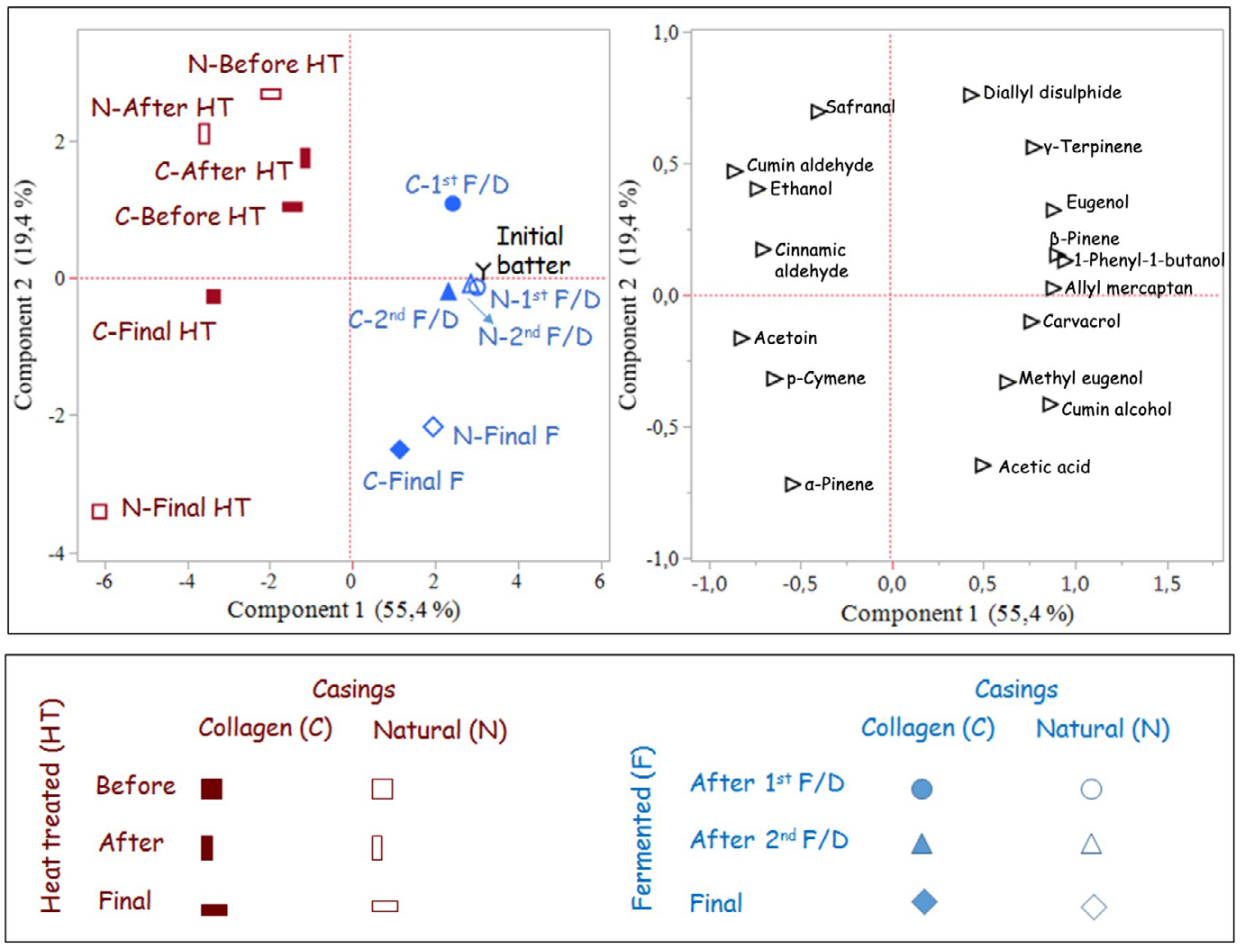
Results of principal component analysis (PCA) of major VCs identified in traditional (blue) and heat treated sucuks (red color) stuffed in to natural (empty symbols) and collagen casing (filled symbols). F/D, fermentation/drying.

The impact of the casing on the volatile compounds during traditional and heat treatment processes was investigated by principal component analysis (PCA), and the results are illustrated in Figure 2. Principal component analysis (PCA) based on eigenvalues showed that the first principal component (PC1) accounted for 55.4% of the total variability, while the second principal component (PC2) explained 19.4%. The PCA bi‐plot demonstrated that the volatile compounds could be utilized to differentiate and categorize the sucuk samples based on their production method and stage, rather than based on the type of casing (Figure 2). PC1 was primarily associated with the production method. Traditional sucuks were located on the right side, while heat‐treated sucuks were positioned on the left side. The traditional sucuks were characterized by a prevalence of acetic acid, cumin alcohol, and methyl eugenol, whereas the heat‐treated sucuks exhibited a dominance of acetoin, p‐cymene, and α‐pinene.

With regard to PC2, the final products showed a distinct positioning compared to the others. The traditional final products were close to each other, whereas the heat‐treated final product with natural casing was far from the collagen casing. The high positive eigenvectors of PC2 were obtained for diallyl disulfide, safranal, γ‐terpinene, ethanol, cumin, and cinnamic aldehyde, which were detected at the highest levels in both the pre‐ and post‐heat‐treated samples. It was also observed that the type of casing provided a more pronounced distinction in heat‐treated sucuks compared to traditional ones. These findings suggest that the impact of casing type on volatile compound differentiation becomes more evident after heat treatment, potentially due to the increased fermentative metabolites present in natural casing. This could explain the distinct positioning of the natural‐cased heat‐treated final product in the PCA (Figure 2), as acetoin, ethanol, and acetic acid were more pronounced in these sucuks (Table 3).

## 4 Conclusion

To the best of our knowledge, this is the first study to report the effect of casing types on volatile compounds in both traditional and heat‐treated sucuks. The present study provided useful information on the optimal casing type for sucuk production according to the production method used. In the traditional method, the type of casing did not have a pronounced impact on the parameters investigated, with the exception of the red color intensity and the browning index value. In both production methods, the red color intensity and the brightness of the collagen‐cased final products were not as satisfactory as those of the natural‐cased ones. The effect of casing type on the volatile compound profile was more pronounced in heat‐treated sucuks than in traditional ones. The heat‐treated final product with natural casing had the highest moisture content, browning index value, cumin aldehyde, p‐cymene, and acetoin, and the lowest γ‐terpinene, eugenol, carvacrol, 1‐phenyl‐1‐pentene, and cumin alcohol. It can be concluded that the use of natural casing in heat‐treated sucuks may not be an optimal choice, as it appears to promote the formation of a greater number of fermentative and oxidative metabolites compared to collagen casing. In light of the present findings, future research will be directed towards investigating the impact of casings on sucuk characteristics, including lipolysis and proteolysis, which are crucial in the development of the final product flavor profile. Furthermore, the use of a starter culture in the heat‐treated process may be recommended to reduce pH and thereby improve product safety.

## Author Contributions

**Ahmet Dursun:** data curation (equal), formal analysis (equal), visualization (equal). **Dilek Türkmen:** data curation (equal), formal analysis (equal). **Zehra Güler:** conceptualization (equal), project administration (equal), writing – review and editing (equal).

## Conflicts of Interest

The authors declare no conflicts of interest.

## Data Availability

The data that support the findings of this study are available from the corresponding author upon reasonable request.
